# Synthetic Seed Technology Development and Production Studies for Storage, Transport, and Industrialization of Bracken Spores

**DOI:** 10.3390/plants9091079

**Published:** 2020-08-22

**Authors:** Bo Kook Jang, Ju Sung Cho, Cheol Hee Lee

**Affiliations:** 1 Division of Animal, Horticultural and Food Sciences, Chungbuk National University, Cheongju 28644, Korea; jangbk@chungbuk.ac.kr (B.K.J.); jsc@chungbuk.ac.kr (J.S.C.); 2 Brain Korea 21 Center for Bio-Resource Development, Chungbuk National University, Cheongju 28644, Korea

**Keywords:** artificial seed, alginate matrix, plug seedling, sporophyte, *Pteridium aquilinum* var. *latiusculum*

## Abstract

Bracken fern (*Pteridium aquilinum* var. *latiusculum* (Desv.) Underw. ex A. Heller) has long been grown industrially in South Korea. Conventional propagation methods, including planting rhizomes and in vitro seedling culture, are labor intensive and expensive, and thus not commercially suitable. We aimed to develop a system to produce synthetic seeds using fern spores (SFS). Synthetic seeds were prepared by mixing bracken spores and alginate matrix. Spore germination and gametophyte and sporophyte growth and development from SFS proceeded normally. Spore density affected gametophyte and sporophyte numbers. SFS prepared using cold (4 °C) long-term storage spores (even 7-year-old spores) could effectively form sporophytes. The highest germination was observed at 25 °C. Soaking-treated SFS successfully formed sporophytes, even after 30 days of storage at 4 °C; indeed, sporophytes formed even after five days of storage at 25 °C during transport conditions. SFS were sown in plug trays for commercial use. Young sporophytes grown from plug seedlings were greenhouse cultivated, and transplanting within eight weeks was effective for root growth and growing-point formation. Developing synthetic seeds is a feasible solution for facilitating efficient transport and the handling of small-sized fern spores; furthermore, this SFS technology provides the basis for fern seedling culture and fern spore industrialization.

## 1. Introduction

Ferns have been consumed as food in many countries for centuries [1]. As rich sources of protein, fiber, minerals, vitamins, essential amino acids, and fatty acids, ferns provide important nutrients to humans [2]. Furthermore, ferns have a high content of secondary metabolites that comprise saccharides, phenolics, polyketides, and terpenoids [3], whose pharmacological activities as diuretics, analgesics, and anti-inflammatories in cases of cystitis, dermatitis, and common cold, and as emmenagogues, have been extensively reported [4,5,6,7]. Bracken fern (*Pteridium aquilinum* var. *latiusculum* (Desv.) Underw. ex A. Heller) is the most popular edible fern in South Korea where, additionally, it has long been used as an industrial crop. Recently, the production of brackens in South Korea has reached 11,043 tons, for an annual revenue of 56 million USD [8], and even more brackens are marketed if imports are taken into account as well. 

The healthiest fern seedlings are best obtained through spore propagation. However, Korean bracken farms normally use a rhizome-cutting propagation method characterized by great difficulty and extremely low efficiency in root growth-point formation, thus requiring intensive labor [9]. Alternatively, a method may be used that consists in sowing spores directly; however, unlike plant seeds, fern spores are very small sized, which makes spore handling in the field difficult, and requires continuous and strict environmental control. Recently, in vitro culture using spores, gametophytes and sporophytes have been attempted, which reduces labor and enables the year-round production of plants [10,11,12,13]. However, in vitro culture requires expensive facilities and equipment, that increase production costs substantially. Further, as knowledge and training in plant tissue culture is required, access to farmers and field use is still limited. 

Alternatively, encapsulation technology might provide a solution to the difficulties in handling fine spores; indeed, the use of synthetic seeds produced by encapsulation technology already offers a solution for plant germplasm regeneration, conservation, storage, and handling [14,15,16]. The biodegradable matrix that constitutes the synthetic seed effectively protects the explants from physical damage and external environment factors; furthermore, it may include nutrients necessary for early growth. Sodium alginate has been widely utilized for its low cost, low toxicity, and gel stability [17]. An insoluble calcium alginate layer is formed by an ion exchange reaction between Na^+^ in the sodium alginate solution and Ca^2+^ in the calcium chloride solution [18]. The production of synthetic seeds has been mainly proposed for the conservation and propagation of endangered species, medicinal and commercial plants including *Tylophora indica* [19], *Curcuma amada* Roxb. [20], *Eclipta alba* [21], *Beta vulgaris* [22], *Erythrina variegata* [16], *Splachnum ampullaceum* moss bud [23], and *Osmunda regalis* gametophyte [24]. Synthetic seed production technology has significantly enhanced the survival rate of explants compared to the existing in vitro culture systems [25,26]. Synthetic seed technology is an inexpensive and practical tissue culture technique and biotechnology.

The present study aimed to develop and produce synthetic seeds for the commercial exploitation of bracken fern. Furthermore, we verified spore germination and the extent of gametophyte and sporophyte development achieved with our new synthetic bracken fern seeds, whose production was intended to solve current problems with the handling, storage, and transportation of bracken spores. Thus, we developed an effective method for the production of bracken seedlings that greatly facilitates spore handling in ex vitro conditions.

## 2. Results

### 2.1. SFS Characteristics

Synthetic seeds produced using bracken spores differed in seed size and weight, depending on the size of the syringe tip. SFS (synthetic seeds using fern spores) produced using the 2.5 mm tip produced an average of 182.0 seeds in 10 mL of alginate solution. SFS were globular, 4.23 mm in diameter, and the mean 50-seed weight was 2610 mg. On the other hand, SFS produced using the 4 mm tip produced an average of 115.3 seeds in 10 mL of alginate solution. In this case, SFS were also globular, but somewhat larger, 5.00 mm in diameter, and the mean 50-seed weight was 4308 mg. 

### 2.2. Effect of SFS on Seed Size and Spore Density

Spore density had the greatest effect on the formation of gametophytes and sporophytes of SFS (Table 1). Gametophyte and sporophyte formation varied significantly with increasing spore density, regardless of SFS size. In particular, the number of gametophytes (3.30), percent sporophyte formation (91.0%), and the number of sporophytes (2.67) were highest for SFS produced using the 2.5 mm tip and a spore density of 1.0 mg/100 mL, although the formation of gametophytes and sporophytes also tended to increase with increasing spore density when the 4 mm tip was used. These results show that the growth of young sporophytes and overall growth improved with increasing spore density (Table 2). On the other hand, SFS size did not affect the growth of young sporophytes.

### 2.3. Effect of Long-Term Cold Storage of Spores on SFS Performance

**Figure 1:**
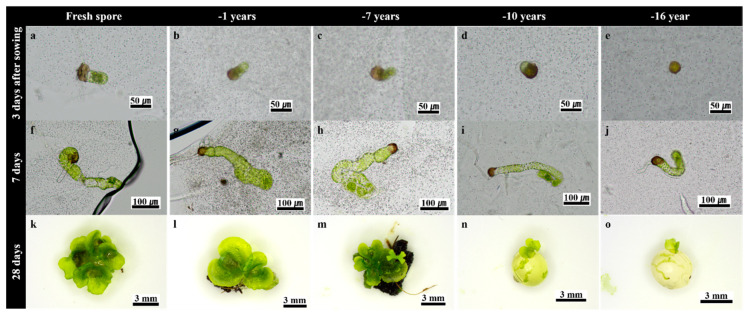
Gametophyte development and response of SFS (synthetic seeds using fern spores) produced with bracken spores stored for different periods at 4 °C: (**a**–**e**) germinated spores 3th day after sowing; (**f**–**j**) germinated spores 7th day after sowing; (**k**–**o**) germinated spores 28th day after sowing.

The results summarized in Table 3 show that bracken spores stored for different periods performed differently with regard to germination, gametophyte development, and sporophyte formation. Thus, spores stored for 10 or 16 years performed poorly, as shown by a significant decrease in the number of gametophytes (>38.7 and 51.2%, respectively), gametophyte size (2.54 and 2.66 mm) and sporophyte formation (30.6 and 23.6%, respectively), compared to fresh spores. On the other hand, more than 80% sporophyte formation (i.e., 1.99–2.38 sporophytes per SFS) was recorded when fresh spores or spores stored for 7 years or 1 year were used to produce bracken SFS. In these cases, gametophyte development rapidly progressed to develop into a filament gametophyte within three days of sowing (Figure 1a–c). Further, cell divisions were observed on the 7th day after sowing, (Figure 1f–h), and on the 28th day after sowing, mature gametophytes were observed on the surface of SFS (Figure 1k–m). On the other hand, the gametophyte development of spores stored for 16 (Figure 1d,i,n) and 10 (Figure 1e,j,o) years was markedly reduced. Furthermore, many gametophytes were observed, both inside and outside of SFS at the initiation of sowing, but after 4 weeks of sowing, between one and four mature gametophytes were observed.

### 2.4. Optimal Temperature for Germination of SFS

Germination of SFS was observed at all temperatures tested; however, spore germination initiation, gametophyte, and sporophyte formation responded differently (Supplemental Table S1). After four weeks, the number of gametophytes observed on the SFS surface at 25 °C was 2.05, over twice the amount observed at the other temperatures tested. In addition, sporophyte formation peaked at 78.47%, and the highest number of sporophytes per SFS was 1.98. SFS sown at 25 °C and 35 °C were regarded as germinated from the second day after sowing, due to the appearance of a rhizoid and chlorophyll cells (Supplemental Figure S1). Healthy looking SFS-gametophytes developed fastest at 25 °C. Conversely, spores took 14 days to germinate when sown under 15 °C and gametophyte development was considerably retarded relative to the 25 °C treatment, while some damage may have caused the interruption of gametophyte development at the higher temperature after 28 days. 

### 2.5. Effect of Packing Conditions for Short-Term Transport of SFS

Synthetic seed soaked and stored at 4 °C showed similar gametophyte (1.94–2.47) and sporophyte formation (71.1–79.2%), regardless of short-term storage duration (Table 4). Thus, even after 30 days of storage, normal germination, gametophyte development and sporophyte formation were observed. SFS short-term storage did not affect sporophyte formation, even when stored without soaking in water (Table 5). Furthermore, high (83.3%) sporophyte formation was obtained, even after a five-day storage period at 25 °C. On the other hand, the number of sporophytes per seed decreased with increasing storage duration. 

### 2.6. Effect of Plug Seedling Period and Transplanting Period on the Growth of SFS Sporophytes

**Figure 2:**
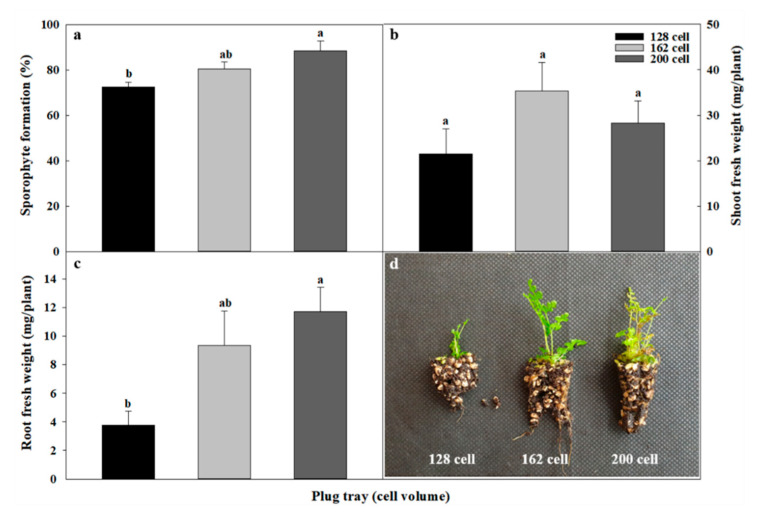
Effect of plug tray size on the growth of bracken SFS (synthetic seeds using fern spores) seedlings (sporophyte). Overall, 128, 162, and 200 cell plug-trays (volume, 21, 15, and 10 cm^3^): (**a**) sporophytes formed in SFS according to plug tray size; (**b**) shoot fresh weight of sporophyte formed in SFS according to plug tray size; (**c**) root fresh weight of sporophyte formed in SFS according to plug tray size; (**d**) growth of sporophytes according to plug tray size 8 weeks after SFS sowing. Different lowercase letters indicate a significant difference at *p* < 0.05 by Duncan’s multiple range test.

The 200 cell-trays allowed excellent root growth, and roots and soil were conveniently densely packed together in the cells (Figure 2). Table 6 summarizes the data on seedling growth as affected by plug seedling period and sporophyte transplanting date. Naturally, young sporophytes cultivated after sowing in a plug-tray showed better growth per individual plant as the cultivation period increased from 8 to 10 and to 12 weeks. However, as shown in Table 6, a sporophyte formed per tray cell is not an individual plant, and as the plug seedling period increased, the number of viable sporophytes decreased. These results influenced the overall growth of sporophyte after transplanting, and plug seedling sporophytes showed poor growth, along with a decrease in viable sporophyte per tray cell on the twelfth week. Furthermore, the root growing-point was not detected in the plug seedling regardless of period. Growing-point formation was observed from the third week after transplanting, and the effect of plug seedling period on the appearance of the root growing-point was negligible. Eight weeks after transplanting, numerous root growing-points were observed.

### 2.7. Sporophyte Production Process by SFS

**Figure 3:**
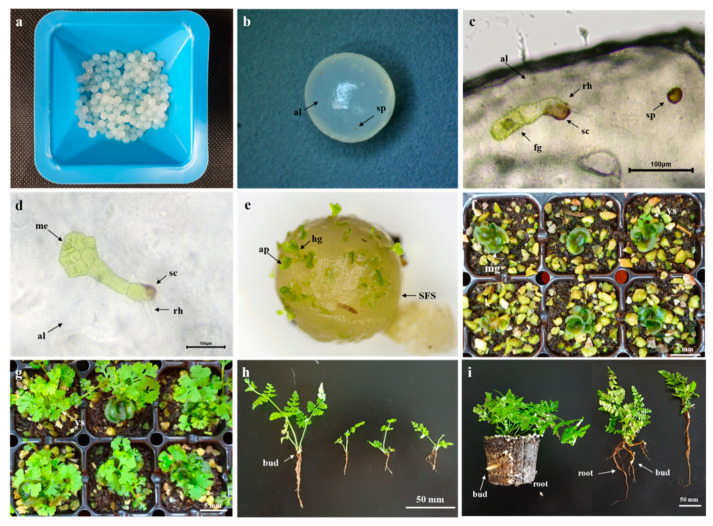
SFS (synthetic seeds using fern spores) production and growth cycle of bracken through spore-derived encapsulation method: (**a**) SFS produced with a 2.5 mm syringe tip; (**b**) spores inserted into the alginate matrix; (**c**) germinated spore and gametophyte after two day in SFS; (**d**), normal developing gametophyte; (**e**) heart-shaped gametophytes formed on the inside and surface of SFS; (**f**) mature gametophytes and young sporophytes developed after 4 weeks in SFS; (**g**) young sporophytes grown after 8 weeks in SFS; (**h**) root growing point observed at three weeks after transplanting; (**i**) strong root development and a large number of root growing-points were observed at eight weeks after transplanting. al, alginate matrix; ap, apical notch; bud, root growing-point; fg, filament gametophyte cell; hg, heart-shaped gametophyte; me, meristematic cell; mg, mature gametophyte; rh, rhizoid; sc, spore coat; sp, spore; ys, young sporophyte.

The sporophyte production process by the produced SFS is shown in Figure 3. SFS was produced with a 1.0 mg/100 mL spore density and a 2.5 mm tip and germinated normally at 25 °C, as in common fern spores, beginning two days after sowing (Figure 3a–e). After four weeks, mature gametophytes were observed (Figure 3f). After eight weeks from SFS sowing (Figure 3g), plug seedlings were successfully produced and transplanted in a greenhouse, where they grew into healthy seedlings with multiple root growing-points (Figure 3h,i). 

## 3. Discussion

The present study aimed to develop a system to produce fern synthetic seed by a spore-derived encapsulation technology using bracken spores. This is the first report to propose the production of synthetic seeds by direct insertion of intact fern spores. These synthetic seeds are intended for planting in an ex vitro environment, not in vitro. Previous studies focused on the production of synthetic seeds based on in vitro culture systems, involving the use of plant growth regulators [14,25,26,27]. In contrast, herein, we report an easily automated, cost-effective system that does not rely on in vitro culture and yet allows large-scale production of bracken seedlings. However, spore density did affect the number of gametophytes and sporophytes. Gametophytes were observed in all spore density treatment groups, but the formation of gametophytes tended to decrease as spore density decreased. The SFSs produced here were globular in shape and 4–5 mm in diameter. SFSs germinated successfully regardless of size. The productivity of the SFS production system design was effectively increased by reducing the syringe tip size used to form the SFS as droplets dripped into the calcium chloride solution. Furthermore, the alginate matrix did not adversely affect spore germination, gametophyte development, or sporophyte formation. The amount of SFS produced was 182.0 per 10 mL (3% alginate matrix and 100 mM calcium chloride), and a maximum production of 300,000 units was predicted if 500 g of alginate matrix was used. Unlike in vitro culture, SFS is easy to produce and economical, because it does not require complex culture facilities and equipment. 

Furthermore, SFS can also be produced using long-term storage spores. The 7-year-old stored spores showed survival rates similar to 1-year-old and fresh spores. In the present study, the germination and the subsequent gametophyte development of 10- and 16-year-old spores were significantly inhibited. The viability of the bracken spores was maintained for 7 years, but decreased continuously thereafter. *Polypodium vulgare* L. spores were also germinated up to 7 years at 4 °C [28]. Although viability decreases as spores age, it can be long preserved at the proper storage temperature [29,30,31]. 

Interestingly, in a few days after sowing, a large number of gametophytes were observed inside and outside the SFS, but only one to four mature gametophytes covered the seed surface four weeks after sowing. Most ferns, except for some species, require light for spore germination [32], and the development of germination and of the gametophyte is sensitive to light [33]. As the earlier gametophytes grew, incident light on the surface of SFS was restricted, whereby the later germinating spores could not develop into a mature gametophyte. These results influenced the number of gametophytes formed per tray cell, and consequently, the number of sporophytes decreased. 

Temperature was an important factor in the germination of SFS. Maximum spore germination, like that of common plant seeds, requires an optimal temperature, which in the case of fern spores, is approximately 25 °C [34]. In the present study, relatively high (35 °C) and low (15 °C) temperatures significantly delayed spore germination and gametophyte development. 

The main goals of SFS production are effortless automated sowing in germination trays and the easy handling of spores. Therefore, our SFS production strategy had to consider short-term storage and transport [16]. SFS tended to have similar germination, gametophyte and sporophyte formation, even after 30 days of storage at 4 °C, if soaked. In addition, vacuum packing conveniently reduced the load weight considered for transportation. SFS stored at 25 °C for five days while in transport formed sporophytes without adverse effects, suggesting that the time required for transport was adequate. Therefore, SFS was able to meet the short-term storage requirement involved in transportation.

Plug trays help to grow seedlings faster after sowing and before transplanting to the soil [35]. The advantage of plug seedling is the cohesion achieved between plant roots and the soil, whereby roots are effectively protected from damage during transplanting [36]. Early sporophyte seedlings form very thin roots and are vulnerable to minor damage. Root growth is very important for sporophyte successful establishment, as new sporophytes are formed from the root growing-points. Therefore, a minimum plug seedling period is required. In the present study, the seeded SFS experienced denser aggregation between soil particles and roots in the smaller tray-cell type. Interestingly, no emergence of the root growing-point was observed during the plug seedling period, and first emergence was observed after transplanting to the greenhouse. Therefore, the cultivation period and the formation of the root growing-point effectively contributed to minimizing the plug seedling period, and thus completed transplant earlier. However, the shoot growth of transplanted sporophytes was not uniform. We speculate that this is because sporophyte growth per tray cell was not uniform. Thus, future research must focus on obtaining uniform sporophyte seedlings.

In conclusion, the production of bracken spore-derived synthetic seeds using alginate matrix and encapsulation technology was successfully developed. Fine spores were converted into seeds to provide the benefits of easy and rapid seed propagation. After plug seedling, transplanted sporophytes formed numerous new root growing-points, but its growth was not uniform. Therefore, research on uniform seedling production should be conducted in future studies. Nevertheless, the SFS production system described greatly reduces production costs and provides an efficient production system with easy handling. Our research provides a sound theoretical basis for solving the problems associated with transport and handling of fern spores, thereby enabling plug seedling and industrialization.

## 4. Materials and Methods

### 4.1. Spore Materials

Mature leaves of *P. aquilinum* were collected in the greenhouse at Chungbuk National University, Cheongju, Korea (36°37′29.1″ N, 127°27′17.1″ E) in September 2018. Leaves were dried in a paper box at 25 ± 1 °C for one week, and then filtered out spores and impurities using a testing sieve (100 μm, Chunggye Sieve, Gunpo, Korea). Collected spores (>one year old) used in the experiments described herein were stored in the dark at 4 °C. Spores kept in storage for 0, 7, 10, and 16 years were used for preparation of SFS (synthetic seeds using fern spores) to investigate the effect of long-term cold storage on spore viability. 

### 4.2. Synthetic Seed Production

The SFS was prepared following the method of [27] with minor modifications. Spores were mixed with 100 mL of 3% (w/v) sodium alginate (CAS 9005-38-3, Duksan Company, Ansan, Korea). Then, the mixture was placed in a 100 mL plastic syringe (Zhejiang Huafu Medical Equipment Co., Ltd., Jiaxing, China) to form the SFS as droplets dripped into a 100 mM CaCl_2_ (CAS 10035-04-8, Daejung Chemicals & Metals Co., Ltd., Siheung, Korea) solution. After 15 min of ion exchange reaction, the CaCl_2_ solution was removed and washed with distilled water.

The germination of SFS and the growth of the seedlings produced by SFS of different sizes obtained by using the syringe with a tip adapter of either 2.5, or 4 mm and different spore densities (0.1, 0.2, 0.5, or 1.0 mg/ 100 mL) were determined. The characteristics of the resulting SFS, i.e., the number of SFS produced per 10 mL of alginate solution, diameter (n = 10), and the 50-seed weight, were investigated. 

SFSs were sown by placing one per cell on a plug-tray containing soil. A 2:1 (v/v) mixture of horticultural substrate and perlite was used to fill plug-trays (162 cells, volume 15 cm^3^) in all experiments, unless otherwise specified. After sowing SFSs, the plug-trays were placed in a box and covered with a glass plate for maintenance of humidity (85 ± 5%) and cultivated at 25 ± 1 °C, under a light intensity of 43 ± 2.0 μmol·m^−2^·s^−1^, and a 16/8 h light/dark regime for eight weeks. Then, all the following experiments were cultivated in the same manner as above, unless otherwise specified.

### 4.3. Determination of Optimal Germination Temperature

Optimal germination temperature (15, 25, 35 °C) of the SFS produced was estimated, and the development of gametophytes and sporophytes was observed, as it is routinely done for intact spore culture [37]. The standard conditions for the production of SFS used in the germination temperature-dependency tests were 1.0 mg/100 mL spore density and 2.5-mm syringe tip-size. 

### 4.4. Short-Term Storage and Transport of SFS

To investigate the effect of short-term cold (4 °C) storage of SFS, after 0, 1, 3, 5, 7, or 30 days, it was stored by soaking in the dark. Produced SFS was soaked in 50-mL conical tubes, which were then completely wrapped in aluminum foil to block the light and stored at 4 °C. In turn, contrasting packing conditions (soakage and vacuum; vacuum packaging machine, JS-777W, JOOnSOO Company, Siheung, Korea), temperatures (4, 25 °C), and three short storage periods (1, 3, or 5 days) were tested to study the effect of transport conditions on SFS. 

### 4.5. Effects of Plug-Tray Cell Volume, Plug Seedling Period and Transplanting Period on Growth of SFS Seedlings

To investigate the effects of the volume of plug-tray cells on plug seedling, SFS was sown in 128-, 162-, and 200-cell plug-trays (21, 15, and 10 cm^3^, respectively). The sporophyte formation and growth according to the plug-tray volume were investigated.

To study the response of sporophyte growth to the duration of plug-seedling period, SFS was sown in 200 cell plug-trays, and then cultivated for 8, 10, or 12 weeks to obtain sporophyte seedlings. Then, seedlings were transplanted to a square pot and cultivated in a greenhouse under 55% shading, for an additional period of 8, 10 or 12 weeks. Sporophyte growth, root growth, and root growing-point formation were compared among culture period treatments. Seedlings were placed completely at random and mist irrigated (tap water) for 5 min, twice every day. For 12 weeks (11 July–8 October 2019), daily average temperature (16.7–32.5 °C) and daily average relative humidity (27.7–79.8%) in the greenhouse were recorded by a data logger (SK-L200TH IIα, skSATO, Tokyo, Japan).

### 4.6. Data Collection and Statistical Analysis

Germinated SFS and gametophyte development were observed using a microscope (CKX53 and SZ61; Olympus, Tokyo, Japan). SFS images were captured using a CMOS camera (eXcope F630; Dixi Sci., Daejeon, Korea) and the eXcope 3.7.12277 software. The number of gametophytes per plug-tray cell was recorded four weeks after sowing, and only gametophytes larger than 3 mm were counted. The success of SFS production was assessed by determining percent sporophyte formation, the number of sporophytes per plug-tray cell, sporophyte fresh weight, leaf length, number of leaves, root length, and number of roots. Four replicates of 27 SFSs were used to investigate seedling production, and four replicates and five plants to assess seedling growth. The same numbers were repeated for all experiments. SAS version 9.4 (SAS Institute Inc., Cary, NC, USA) was used to calculate mean ± standard error for each treatment, and a factorial analysis was performed using Duncan’s multiple range test at *p* < 0.05.

## Figures and Tables

**Table 1:** Effect of syringe tip size and spore density on gametophyte and sporophyte formation from bracken SFS (synthetic seeds using fern spores).

Syringe Tip Size	Spore Density (mg/100 mL)	No. of Gametophytes/SFS Seed	Sporophyte Formation (%)	No. of Sporophytes/SFS Seed
2.5 mm	0.1	0.85 ± 0.10 b	27.8 ± 4.7 fg	1.19 ± 0.08 e
	0.2	1.41 ± 0.10 b	61.8 ± 4.3 cd	1.88 ± 0.10 cd
	0.5	2.65 ± 0.12 a	69.4 ± 3.6 bc	1.98 ± 0.14 bc
	1.0	3.30 ± 0.24 a	91.0 ± 1.3 a	2.67 ± 0.20 a
4 mm	0.1	1.07 ± 0.09 b	16.7 ± 5.2 g	1.18 ± 0.07 e
	0.2	1.48 ± 0.23 b	42.4 ± 6.1 ef	1.55 ± 0.11 de
	0.5	2.52 ± 0.05 a	52.8 ± 9.4 de	1.29 ± 0.11 e
	1.0	3.00 ± 0.82 a	78.5 ± 1.7 ab	2.29 ± 0.08 b
Significance ^z^				
Size (A)		NS	***	***
Density (B)		***	***	***
A × B		NS	NS	NS

Different lowercase letters within each column indicate a significant difference at *p* < 0.05 by Duncan’s multiple range test. ^z^ NS, not significant; ***, significance at *p* = 0.001.

**Table 2:** Effect of syringe tip size and spore density on the growth of sporophytes cultured from bracken SFS (synthetic seeds using fern spores).

Syringe Tip Size	Spore Density (mg/100 mL)	No. of Leaves/Plant	Leaf Length (mm)	No. of Roots/Plant	Root Length (mm)	Shoot F.W. (mg/Plant)	Root F.W. (mg/Plant)
2.5 mm	0.1	2.70 ± 0.13 b	15.78 ± 1.25 cd	2.95 ± 0.26 bc	24.98 ± 3.30 a	10.61 ± 1.96 bc	2.28 ± 0.80 bc
	0.2	3.40 ± 0.14 a	18.02 ± 0.81 b–d	3.75 ± 0.26 ab	26.20 ± 4.04 a	14.77 ± 1.05 ab	3.42 ± 0.71 bc
	0.5	3.55 ± 0.17 a	20.17 ± 0.78 a–c	4.30 ± 0.30 a	29.42 ± 1.50 a	18.93 ± 2.11 ab	4.70 ± 0.66 ab
	1.0	3.35 ± 0.21 a	21.84 ± 2.62 ab	3.70 ± 0.40 ab	25.62 ± 5.30 a	23.47 ± 6.25 a	4.23 ± 1.62 ab
4 mm	0.1	2.45 ± 0.05 b	13.36 ± 2.07 d	2.20 ± 0.18 c	13.46 ± 3.35 b	4.13 ± 0.35 c	0.41 ± 0.11 c
	0.2	3.80 ± 0.18 a	23.94 ± 0.97 a	4.05 ± 0.25 a	34.58 ± 2.18 a	25.69 ± 3.78 a	7.32 ± 1.24 a
	0.5	3.45 ± 0.10 a	22.52 ± 1.63 ab	3.75 ± 0.21 ab	31.39 ± 3.32 a	18.40 ± 2.16 ab	4.80 ± 1.16 ab
	1.0	3.70 ± 0.13 a	22.21 ± 2.42 ab	3.85 ± 0.17 a	30.02 ± 2.51 a	23.43 ± 4.80 a	5.59 ± 1.36 ab
Significance ^z^							
Size (A)		NS	NS	NS	NS	NS	NS
Density (B)		***	***	***	**	***	**
A × B		NS	NS	NS	*	NS	NS

Different lowercase letters within each column indicate a significant difference at *p* < 0.05 by Duncan’s multiple range test. ^z^ NS, not significant; *, **, and ***, significance at *p* = 0.05, 0.01, and 0.001, respectively.

**Table 3:** Sporophyte formation and growth of SFS (synthetic seeds using fern spores) produced with bracken spores stored for different periods at 4 °C.

Spore Storage (Years)	No. of Gametophytes/SFS Seed	Wings Width of Gametophyte (mm)	Sporophyte Formation (%)	No. of Sporophytes/SFS Seed	No. of Leaves/Plant
−16	1.16 ± 0.09 e	2.66 ± 0.33 b	23.6 ± 5.01 b	1.38 ± 0.07 b	3.67 ± 0.24 b
−10	1.46 ± 0.09 d	2.54 ± 0.20 b	30.6 ± 2.78 b	1.31 ± 0.10 b	3.60 ± 0.12 b
−7	1.86 ± 0.09 c	4.32 ± 0.35 a	81.9 ± 3.67 a	2.38 ± 0.14 a	4.33 ± 0.13 a
−1	2.13 ± 0.06 b	5.11 ± 0.36 a	80.6 ± 6.05 a	1.99 ± 0.15 a	4.27 ± 0.13 a
0	2.38 ± 0.03 a	4.69 ± 0.26 a	84.7 ± 5.56 a	2.33 ± 0.12 a	4.33 ± 0.13 a
**Spore Storage (Years)**	**Leaf Length (mm)**	**No. of Roots/Plant**	**Root Length (mm)**	**Shoot FW (mg/Plant)**	**Root FW (mg/Plant)**
−16	19.66 ± 3.60 c	3.92 ± 0.42 c	26.90 ± 4.06 a	21.59 ± 5.66 b	4.15 ± 1.11 a
−10	22.69 ± 1.06 c	4.47 ± 0.07 c	29.66 ± 1.62 a	23.70 ± 2.24 b	4.03 ± 0.50 a
−7	29.78 ± 1.12 b	4.73 ± 0.18 bc	31.65 ± 4.88 a	29.44 ± 3.12 ab	4.06 ± 0.32 a
−1	34.42 ± 1.84 ab	5.80 ± 0.23 a	32.29 ± 3.02 a	35.36 ± 4.94 ab	6.51 ± 1.19 a
0	36.99 ± 1.86 a	5.67 ± 0.48 ab	35.90 ± 2.05 a	40.26 ± 3.64 a	6.95 ± 1.14 a

Different lowercase letters within each column indicate a significant difference at *p* < 0.05 by Duncan’s multiple range test.

**Table 4:** Effect of short-term storage of SFS (synthetic seeds using fern spores) at 4 °C on sporophyte formation and growth.

Packing (Days)	No. of Gametophytes/SFS Seed	Sporophyte Formation (%)	No. of Sporophytes/SFS Seed	No. of Leaves/Plant	
1	1.94 ± 0.14 a	79.2 ± 4.17 a	1.80 ± 0.11 a	3.83 ± 0.08 a	
3	2.05 ± 0.06 a	73.2 ± 3.15 a	1.69 ± 0.06 a	3.40 ± 0.12 b	
5	2.47 ± 0.34 a	72.2 ± 4.01 a	1.72 ± 0.12 a	3.33 ± 0.18 b	
7	2.13 ± 0.17 a	74.4 ± 9.49 a	2.18 ± 0.27 a	3.87 ± 0.13 a	
30	2.19 ± 0.25 a	71.1 ± 5.88 a	1.95 ± 0.13 a	3.67 ± 0.08 ab	
**Packing (Days)**	**Leaf Length (mm)**	**No. of Roots/Plant**	**Root Length (mm)**	**Shoot FW (mg/Plant)**	**Root F.W. (mg/Plant)**
1	25.67 ± 1.36 a	4.75 ± 0.14 a	29.38 ± 2.08 a	21.43 ± 1.66 a	3.23 ± 0.55 ab
3	19.46 ± 1.66 b	3.40 ± 0.23 c	24.73 ± 1.49 a	12.42 ± 1.06 b	1.83 ± 0.11 b
5	17.30 ± 0.24 b	3.73 ± 0.07 bc	22.28 ± 3.04 a	13.80 ± 0.67 b	2.51 ± 0.14 b
7	26.32 ± 1.82 a	4.47 ± 0.35 ab	25.38 ± 2.89 a	19.90 ± 3.05 a	2.88 ± 0.43 ab
30	24.48 ± 0.89 a	4.25 ± 0.25 ab	22.37 ± 3.24 a	20.99 ± 0.68 a	4.15 ± 0.63 a

Different lowercase letters within each column indicate a significant difference at *p* < 0.05 by Duncan’s multiple range test.

**Table 5:** Effect of packing conditions for short-term storage and transport on gametophyte and sporophyte formation from bracken SFS (synthetic seeds using fern spores).

Packing Condition			No. of Gametophytes/SFS Seed	Sporophyte Formation (%)	No. of Sporophytes/SFS Seed
Types	Temp. (°C)	Period (Days)			
Soakage	4	1	2.97 ± 0.15 ab	88.9 ± 5.01 ab	2.24 ± 0.14 ab
		3	2.44 ± 0.05 c–e	86.1 ± 7.73 a–c	2.14 ± 0.12 b
		5	1.79 ± 0.15 f	76.4 ± 1.39 cd	1.58 ± 0.04 c
	25	1	3.19 ± 0.14 a	94.4 ± 2.78 ab	2.64 ± 0.09 a
		3	2.33 ± 0.10 de	88.9 ± 3.67 ab	2.18 ± 0.07 b
		5	2.70 ± 0.13 a–d	93.1 ± 3.67 ab	2.10 ± 0.22 b
Vacuum	4	1	2.95 ± 0.14 a–c	94.4 ± 2.78 ab	2.08 ± 0.08 b
		3	1.97 ± 0.29 ef	76.4 ± 1.39 cd	1.89 ± 0.11 bc
		5	1.54 ± 0.21 f	65.3 ± 2.78 d	1.62 ± 0.16 c
	25	1	3.11 ± 0.15 ab	91.7 ± 2.41 ab	2.24 ± 0.27 ab
		3	2.62 ± 0.15 b–d	98.6 ± 1.39 a	2.14 ± 0.15 b
		5	2.40 ± 0.18 de	83.3 ± 4.17 bc	1.88 ± 0.05 bc
Significance ^z^					
Packaging (A)			NS	NS	NS
Temperature (B)			***	***	**
Period (C)			***	***	***
A × B			NS	NS	NS
A × C			NS	NS	NS
B × C			*	*	NS
A × B × C			NS	*	NS

Different lowercase letters within each column indicate a significant difference at *p* < 0.05 by Duncan’s multiple range test. ^z^ NS, not significant; *, **, and ***, significance at *p* = 0.05, 0.01, and 0.001, respectively.

**Table 6:** Effect of plug-seedling period and transplanting period on growth and root growing-point formation in bracken SFS (synthetic seeds using fern spores) seedlings.

Plug ^z^ (Weeks)	Trans. ^y^ (Weeks)	Sporophyte Survival/Cell	Total Bud ^x^ Number/Cell	Total Root Number/Cell	Root Length (cm)	Root FW (g/Plant)
8	8	2.00 ± 0.32 ab	4.60 ± 0.40 e	56.00 ± 4.42 b–d	10.86 ± 1.52 de	0.91 ± 0.19 c
	10	2.20 ± 0.20 ab	11.80 ± 0.80 bc	79.00 ± 10.02 bc	14.52 ± 2.05 a–d	2.69 ± 0.52 ab
	12	2.50 ± 0.29 a	21.00 ± 2.38 a	127.75 ± 17.25 a	16.45 ± 0.88 ab	3.89 ± 0.55 a
10	8	2.50 ± 0.65 a	5.75 ± 0.75 de	84.75 ± 8.63 b	9.44 ± 1.40 e	1.36 ± 0.55 bc
	10	2.00 ± 0.00 ab	15.75 ± 3.09 b	65.50 ± 13.93 b–d	13.19 ± 0.76 b–e	2.64 ± 0.34 ab
	12	2.75 ± 0.25 a	21.25 ± 1.89 a	126.75 ± 6.71 a	15.73 ± 2.06 a–c	3.84 ± 0.64 a
12	8	2.20 ± 0.37 ab	8.00 ± 2.16 c–e	47.75 ± 5.36 d	11.43 ± 1.35 c–e	1.42 ± 0.25 bc
	10	1.20 ± 0.20 b	10.75 ± 1.11 b–d	53.25 ± 5.02 cd	19.15 ± 1.53 a	2.69 ± 0.61 ab
	12	1.20 ± 0.20 b	13.00 ± 2.04 bc	61.00 ± 2.94 b–d	18.36 ± 0.75 a	2.74 ± 0.47 ab
Significance ^w^						
Sowing (A)		***	NS	***	*	NS
Seedling (B)		NS	***	***	***	***
A × B		NS	*	*	NS	NS

Different lowercase letters within each column indicate a significant difference at *p* < 0.05 by Duncan’s multiple range test. ^z^ Young sporophyte obtained after sowing, according to the period of plug seedling. ^y^ The sporophyte grown after transplanting the young sporophyte obtained from the plug seedling into the greenhouse for each period. ^x^Root growing-point. ^w^ NS, not significant; *, and ***, significance at *p* = 0.05, and 0.001, respectively.

## Supplementary Materials

The following are available online at https://www.mdpi.com/2223-7747/9/9/1079/s1, Table S1: Effect of different germination temperatures on SFS (synthetic seeds using fern spores) of bracken, Figure S1: Gametophyte development and response of bracken SFS (synthetic seeds using fern spores) to different temperature. ap, apical notch; fg, filament gametophyte cell; hg, heart-shaped gametophyte; me, meristematic cell; rh, rhizoid; sc, spore coat.
