# Supplementary material for: Synthetic Seed Technology Development and Production Studies for Storage, Transport, and Industrialization of Bracken Spores
Source: Plants (Basel). 2020 Aug 22;9(9):1079. doi: 10.3390/plants9091079 (PMC7570132; doi:10.3390/plants9091079)
Supplement: Supplementary file 1 [file plants-09-01079-s001.pdf]

Supplemental Table S1. Effect of different germination temperatures on SFS (synthetic seeds using fern spores) of bracken.

Temperature (°C)	No. of gametophytes/ SFS[z] seed	Sporophyte formation (%)	No. of sporophytes/ SFS seed
15	0.96 ± 0.08 b	0.00 ± 0.00 b	0.00 ± 0.00 c
25	2.05 ± 0.17 a	78.47 ± 2.50 a	1.98 ± 0.15 a
35	0.79 ± 0.08 b	6.94 ± 1.84 b	1.00 ± 0.00 b

Different lowercase letters within each column indicate a significant difference at $P < 0.05$ by Duncan's multiple range test.

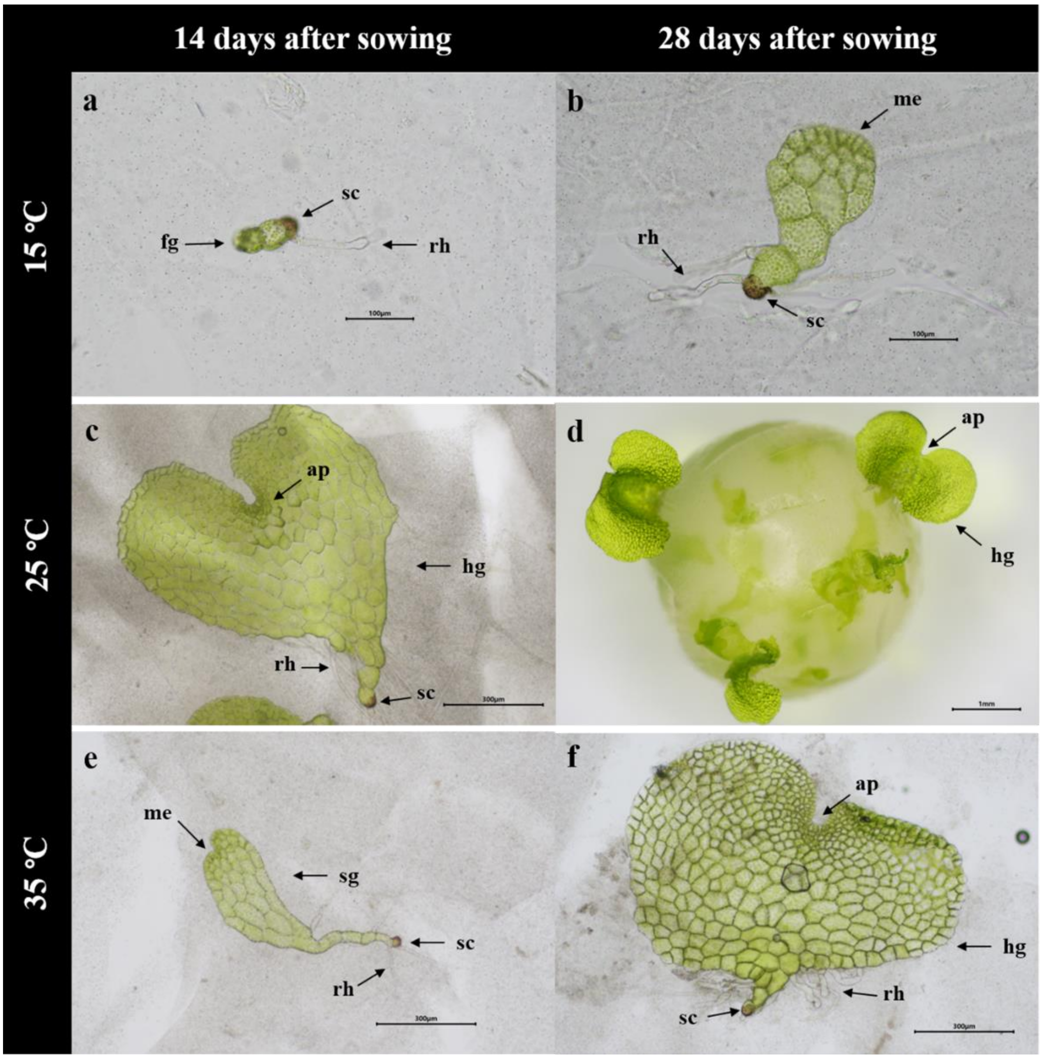

Supplemental Fig. S1. Gametophyte development and response of bracken SFS (synthetic seeds using fern spores) to different temperature. ap, apical notch; fg, filament gametophyte cell; hg, heart-shaped gametophyte; me, meristematic cell; rh, rhizoid; sc, spore coat.
